# Development and validation of a quality of life questionnaire for patients with colostomy or ileostomy

**DOI:** 10.1186/1477-7525-3-62

**Published:** 2005-10-12

**Authors:** Luis Prieto, Hanne Thorsen, Kristian Juul

**Affiliations:** 1 Health Outcomes Consultant. C/ Rioja, 7. 28750 San Agustin de Guadalix, Spain; 2 Institute of Public Health, Department of General Practice, University of Copenhagen, Denmark; 3 Ostomy Division, Clinical Documentation Department, Coloplast A/S, Holtedam 1, DK-3050 Humlebæk, Denmark

**Keywords:** Stoma, quality of life, development, validation, reliability, Rasch analysis

## Abstract

**Background:**

Quality of life of stoma patients is increasingly being addressed in clinical trials. However, the instruments used in the majority of these studies have not been validated specifically for stoma patients. The aim of this paper is to describe the development and validation of a quality-of-life instrument, "Stoma-QOL", specifically for patients with colostomy or ileostomy.

**Methods:**

Potential items were formulated in English on the basis of the results of a series of semi-structured interviews with 169 adult stoma patients. The process resulted in a preliminary 37-item version, which was translated into French, German, Spanish and Danish, and administered repeatedly to 182 patients with colostomy or ileostomy. A psychometric selection of items was performed through Rasch Analysis. The measurement properties of the final questionnaire version were subsequently tested.

**Results:**

The 20 items in the final questionnaire covered four domains – sleep, sexual activity, relations to family and close friends, and social relations to other than family and close friends. These items were found to define a unidimensional variable according to Rasch specifications (Infit MNSQ < 1.3). Internal consistency reliability calculated as Cronbach's alpha was 0.92, i.e., highly reliable. Spearman's correlation coefficients of scores across times of administration was >0.88 (p < 0.01), indicating a high test-retest reliability. Item calibrations by country calculated as ICC were 0.81 (0.67–0.91 95% CI), confirming cross-cultural comparability across the European countries included in the study.

**Conclusion:**

Given the adequacy of the metric properties of the Stoma-QOL suggested by the psychometric analyses, this study confirms the suitability of the instrument in clinical practice and in clinical research.

## Background

Stoma patients have a surgically created opening on the abdomen involving parts of either the gastrointestinal or urinary tract. Colostomy involves discharging feces from the large intestine, ileostomy from the small intestine, while urostomy means discharging urine through the surgical opening. Due to this major change in physical appearance and bodily function, patients with stoma are challenged with a number of quality of life (QOL) issues.

In recent years QOL of stoma patients has been addressed in a number of studies [1-6], some covering a broad range of different stomas, others focusing more narrowly on just one or two of the conditions – colostomy, ileostomy or urostomy.

With few exceptions, the abovementioned instruments were not reported to have been validated specifically for stoma patients. Since the development and validation of Olbrisch's "Ostomy Adjustment Scale" in the early 1980's [1], to our knowledge the only contemporary instrument constructed and psychometrically tested specifically for colo-, ileo- and urostomy has been the "Stoma Care Quality of Life Index" (1998) [6]. This 34-item questionnaire was validated in the UK and France, showing a satisfactory reliability.

However, the psychometric properties of both the "Ostomy Adjustment Scale" [1] and the "Stoma Care Quality of Life Index" [6] were assessed solely within a classical theoretical approach, the so-called Classical Test Theory [7], which is a valid method, but in our opinion may not be the optimal solution. The main problem with the Classical Test Theory is that it presupposes that one can directly infer, e.g., a stoma patient's quality of life by summing responses and calculating a total score, assuming that each item contributes equally to this total score. However, treating items equally implies that all items are of identical importance, which in our experience might not be the case. A stoma patient's strong agreement with an item like "I worry that my family feel awkward around me" indicates a greater problem than does a strong agreement with an item like "I become anxious when the pouch is full". Thus, when items represent different levels of importance to the stoma patients' quality of life, should the data not be analysed so that the total score reflects this value of "importance" of the item's contribution to the total scale value?

To address this question, which to our knowledge has never been addressed before in association with measurements of quality of life in stoma patients, our aim was to develop a simple, cross-cultural and reliable measurement of quality of life in stoma patients, "Stoma-QOL", and to validate this instrument according to both the Classical Test Theory and the modern Item Response Theory [8], thereby taking into account the "importance" weight of each item in the test (see Methods section for details).

## Methods

### The psychometric models used in the development and validation of Stoma-QOL

The content of the new stoma-specific QOL instrument was developed on the basis of Hunt and McKenna's needs-based model of QOL [9]. This model draws on the work of theorists in the field of human motivation who postulate that individuals are motivated or driven by their needs, e.g., as defined in Maslow's well-known hierarchy of needs pyramid [10]. For this study, this approach implied that rather than relying on literature or experts to determine needs important to patients, the original content of the questionnaire was derived from qualitative interviews with stoma patients.

For the reasons briefly mentioned in the Introduction, the Item Response Theory [8] was our primary model for analysis of the questionnaire resulting from the interviews with stoma patients. This method is built around the idea that the probability of a patient's answer when confronted with a certain item ideally can be described as a simple function of the patient's position on a latent trait (e.g., quality of life) plus one or more parameters characterising the particular item (e.g., its "severity" or "importance" or "weight").

The Item Response Theory [8]*measures *the quality of a given test as a measurement instrument, and helps to predict its performance in future applications. However, the Item Response Theory can also assist in *improving *the quality of the test, e.g. by indicating which items are inappropriate and should be changed, deleted, or replaced. After this process, the test can be used as a standard instrument to measure similar patients. Skipping mathematical explanations, the Item Response Theory can do more things than the Classical Test Theory [7,8] when it comes to modelling existing tests, constructing new ones, and, above all, interpreting the results of measurement.

We chose the Rasch model [11,12] as this is a simple but at the same time very powerful Item Response Theory model for measurement. The Rasch model uses the traditional total score (i.e., the sum of the item ratings) as a starting point for estimating response probabilities. The model is based on the simple idea that some items are more important to patients than other items. Thus, the Rasch model constructs a line of measurement with the items placed hierarchically on this line according to their importance to patients. The validity of a given test can be assessed through examination of this item ordering, i.e. by assessing whether all items work together to measure a single variable.

See the section "Analysis and item reduction of the 37-item questionnaire" for details on the practical implementation of these theoretical models.

### Development of the Stoma-QOL questionnaire

**Figure 1:**
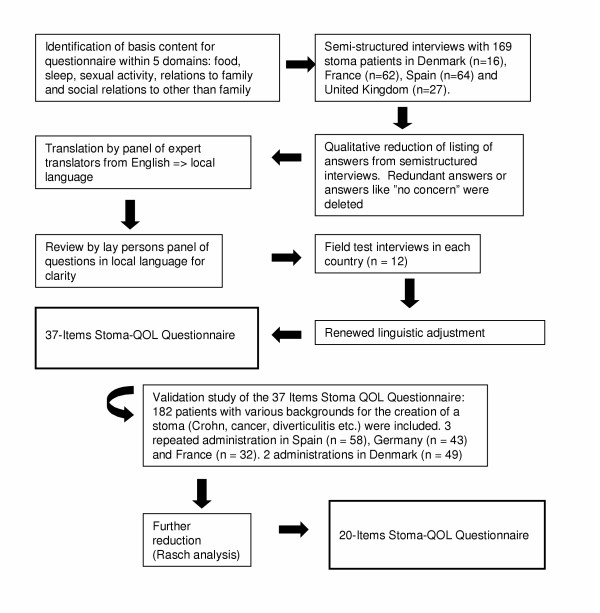
Development of the Stoma-QOL questionnaire.

Figure 1 details the process of the cross-cultural development of the Stoma-QOL questionnaire.

The development was initiated by the formulation of potential stoma-related items in English on the basis of the results of a series of semi-structured interviews conducted by stoma care nurses with 169 stoma patients in France, Denmark, Spain and the United Kingdom.

The interviews were structured to cover the following five broad domains, which are included in Maslow's hierarchy of needs pyramid [10] and at the same time were supported by the experience of stoma care nurses in their daily routine with stoma patients:

1. What, if any, concerns do you have about what you can eat?

2. What, if any, concerns do you have about sleeping?

3. What, if any, concerns do you have about intimate relations?

4. What, if any, concerns do you have regarding your relationship with family and close friends?

5. What, if any, concerns do you have regarding your relationship with people other than family and close friends?

Stoma care nurses put these questions to the patients in their respective national languages, and the answers were collected on a special form. Answers given in non-English-speaking countries were translated into English. A common listing of the answers was generated from these interviews. Redundancies and answers like "no concerns" were removed.

The next step consisted in the selection, at a meeting between the national investigators, of items that could be translated from English into the four non-English languages involved in the project (German, Spanish, French and Danish). It was ensured that all the items that were chosen were consistent with the need-based theory of quality of life [9]. Furthermore, it was decided that the items should be formulated so that they could be meaningfully answered with the following four response categories: "Always", "Sometimes", "Rarely" and "Not at all".

Following an international accepted protocol [13], the translation of the questionnaire from English into the four non-English languages proceeded in three steps. First, the items were translated by a panel of bilingual translators. In the second step, this intermediate translation was assessed by a panel of lay persons for linguistic clarity, understandability and easiness to complete. Thirdly, field tests were conducted as individual interviews with 12 stoma patients in each country, after which, where necessary, items were again semantically adjusted without distorting the content of the items. The process resulted in a 37-item translated version for each country.

Finally, a specific validation study was initiated in each country, aiming to test the psychometric properties of the preliminary 37-item questionnaire. This study also aimed at reducing the number of items through psychometric analysis. We wanted to preserve as much as possible of the structure of the preliminary questionnaire and, in addition, to allow for calculation of one global score. The measurement properties of the final 20-item questionnaire were subsequently tested as described in the following section.

### Analysis and item reduction of the 37-item questionnaire

The original 37-item questionnaire was analysed with the Rasch Rating Scale model in a special version allowing more than two answer categories to be modelled for each item for the overall sample. Rasch analyses were performed with Version 3.0.1 of the WINSTEPS computer program [14]. An item calibration was obtained for each item.

In order to determine how well each item contributed to common global health measurement, chi-square fit statistics, known as Infit Mean Square (Infit MNSQ), were also calculated [12]. In this analysis values greater than 1.3 imply a potential misfit to the Rasch model [15], and items with values above this threshold were consequently removed from the test. Successive Rasch analyses were performed until all the remaining items showed acceptable goodness-of-fit properties.

Subsequently, the performance of the final 20-item questionnaire was determined as the index of person separation (PSEP) [7,8,12]. The index of person separation describes how reliably the patients are separated by the scale and has to exceed 2 (or 3) in order to confirm an optimal level of reliability of 0.80 (or 0.90).

Stratified Rasch analyses were also performed for each country in the study. The concordance of item calibrations by country was assessed through an Intraclass Correlation Coefficient (ICC) [15]. This is a statistical procedure used to determine the reproducibility of a measurement of a variable. The ICC is based on variance components analysis and measures the homogeneity within groups relative to the total variation. The ICC is large when there is little variation within the groups compared to variation among group means, where groups consist of replicate measurements. A small ICC occurs when within-group variation is large compared with between-group variability, indicating that some unknown variable has introduced nonrandom effects in the different groups. The maximum value of the ICC is 1, and the minimum value is theoretically 0. The ICC is routinely used in epidemiological studies to address the test-retest reliability, validity of questionnaires and interlaboratory concordance.

As a secondary model for analysis, the final 20 items were also subjected to a traditional item analysis. We used the following gold standard statistical procedures based on Classical Test Theory [8]: a) classical index of discrimination was calculated to measure the spread of scores between the patients; b) difficulty indices were determined by calculating the mean response choice for each item; c) Cronbach's alpha coefficient was calculated to estimate internal-consistency; d) Exploratory Factor Analysis (EFA) was performed in order to test the unidimensionality of the reduced version; e) test-retest reliability estimates were obtained for the reduced scale by calculating Spearman's coefficients of correlation across the different times of administration of the questionnaire (T1, T2 and T3); and f) distribution patterns of scores were described for each reduced questionnaire, overall and by country.

The Statistical Package for Social Sciences (SPSS), version 10, was used to perform all the above analyses.

## Results

### Patients

One hundred and eighty-two patients from four different European countries with various backgrounds for the creation of a stoma (Crohn's, cancer, diverticulitis, etc.) were included in the validation study Mean age was 53 years ranging from 18 to 84 years, with slightly more males than females (Table 1). 52% had colostomy and 48% had ileostomy. No urostomy patients were included. All patients were in a stable period or cured, with a duration since stoma creation ranging from 0 to 43 years, when they participated in the study. Incomplete or missing data for up to 12 patients resulted in a sample less than 182 on some of the abovementioned demographic variables.

**Table 1:** Demographics of patients in the validation study

	**DK**	**Germany**	**Spain**	**France**	**Total**
N*)	49	43	58	32	182
Age (years), mean (SD)	58.2 (12.5)	61.9 (11.9)	40.7 (11.6)	58.5 (14.5)	53.4 (15.3)
Sex (Male/Female), N (%)	14/32 (30%/70%)	33/8 (80%/20%)	28/30 (48%/52%)	18/14 (56%/44%)	93/84 (52.5%/47.5%)
Type of stoma					
Colostomy, N (%)	23 (50.0%)	34 (100.0%)	0 (0.0%)	32 (100.0%)	89 (52.4%)
Ileostomy, N (%)	23 (50.0%)	0 (0.0%)	58 (100.0%)	0 (0.0%)	81 (47.6%)
Duration from stoma creation (years), mean (SD)	15.4 (11.0)	4.8 (3.4)	4.6 (4.8)	2.7 (5.4)	7.1 (8.4)

*) Missing demographic data for up to N = 12 means that sample N will be less than 182 on some variables. Means, SD and percentages refer to valid cases only.

Responses to the 37-item questionnaire came from two different study sources. The patients from France, Germany and Spain were included as a part of controlled clinical trials (randomised cross-over designs) conducted to test a new stoma pouch. Where required these trials were approved by ethics committees and informed consent was obtained. In accordance with the protocol of the clinical trials, the patients responded to the questionnaire on three different occasions. The patients from Denmark were recruited directly through a stoma patient database approved by the Danish Data Protection Agency. For logistic reasons, these patients did not test any stoma products and only responded twice to the instrument.

### Results from psychometric analysis

The overall Rasch analysis of the 37 items of the original questionnaire (Table 2) showed 6 misfitting items. Infit MNSQ statistics ranged from 0.69 to 1.40 (SD = 0.19). Misfitting items in this and subsequent analyses were removed until no further improvement in fit requirements was found. Seventeen items (items 1, 2, 6, 7, 9, 10, 11, 12, 14, 15, 20, 28, 30, 31, 32, 36 and 37) were discarded in this process (performed in seven different steps), reducing the initial questionnaire to 20 items, the final Stoma-QOL. During the process all questions belonging to the domain related to food were omitted because these items did not contribute to constructing, with the remaining items, a common and single health-related quality of life variable. With four response choices per question (1. Always; 2. Sometimes; 3. Rarely; 4. Not at all), the highest possible raw score for the reduced questionnaire is 80 (best QOL) and the lowest possible score is 20 (worst QOL).

**Table 2:** Content of the Original 37-Item Questionnaire and of the Final, Reduced Version, Stoma-QOL (4 response choices: 1-Always, 2-Sometimes, 3-Rarely, 4-Not at all)

**Original 37-Item Questionnaire**	**Stoma-QOL + : item included - : item excluded**
1. I worry about skin problems where the pouch attaches	-
2. Because of my stoma I prefer eating at home	-
3. I feel the need to know where the nearest toilet is	+
4. I become anxious when the pouch is full	+
5. I feel tired during the day	+
6. I worry that my family will reject me	-
7. I avoid sexual intimacy because of my stoma	-
8. I am afraid of meeting new people	+
9. I am preoccupied by what I can eat and drink	-
10. I worry that friends will reject me	-
11. My sleep is interrupted because of my stoma	-
12. I avoid sleeping in certain positions	-
13. It is difficult to hide the fact that I wear a pouch	+
14. I have to avoid drinks that I like	-
15. I have problems falling asleep	-
16. My stoma makes it difficult for me to be with other people	+
17. I sleep badly during the night	+
18. I feel lonely even when I am with other people	+
19. I need to rest during the day	+
20. I worry about the pouch leaking	-
21. I worry that my condition is a burden to people close to me	+
22. I avoid close physical contact with my friends	+
23. I worry that my family feel awkward around me	+
24. I feel embarrassed about my body because of my stoma	+
25. It would be difficult for me to stay away from home overnight	+
26. I worry that the pouch rustles	+
27. I worry that the pouch may smell	+
28. I am afraid of being rejected sexually because of my stoma	-
29. My stoma makes me feel sexually unattractive	+
30. I worry that my friends feel awkward around me	-
31. I have to think about my pouch when planning my day	-
32. I avoid close physical contact with my family	-
33. I worry about noises from the stoma	+
34. I worry that the pouch will loosen	+
35. My stoma pouch limits the choice of clothes that I can wear	+
36. I have to avoid situations where I over-perspire (for example, brisk walking or sports)	-
37. I avoid getting changed in front of other people	-

There were 178 individuals (out of 182) susceptible to measurement in the Rasch analysis. A total of four cases were not considered in the analysis, since they reported a maximum extreme score (n = 2), or lacked responses for the whole questionnaire (n = 2). Valid responses accounted for 98.3% of the sample. The item calibrations, or the item "weights", varied from -1.60 to 1.33 logits, all but one below the threshold of 1.3 for a potential misfit. The 20 items fit to define a unidimensional variable according to initial Rasch specifications (Infit MNSQ < 1.3) (Table 3).

**Table 3:** Rasch Analysis of the Items of the Final, Reduced Stoma-QOL: Item Statistics by Measure (or Item Calibration) Order.

Item no.	Item text	Calibration	SE	Infit MNSQ
i4	I become anxious when the pouch is full	1.33	0.10	0.78
i34	I worry that the pouch will loosen	1.16	0.10	1.13
i3	I feel the need to know where the nearest toilet is	1.02	0.10	1.16
i27	I worry that the pouch may smell	0.92	0.10	1.05
i33	I worry about noises from the stoma	0.72	0.09	0.88
i19	I need to rest during the day	0.42	0.09	0.93
i35	My stoma pouch limits the choice of clothes that I can wear	0.35	0.10	1.07
i5	I feel tired during the day	0.27	0.09	0.80
i29	My stoma makes me feel sexually unattractive	0.23	0.11	1.22
i17	I sleep badly during the night	0.08	0.10	1.16
i26	I worry that the pouch rustles	-0.03	0.10	1.19
i24	I feel embarrassed about my body because of my stoma	-0.10	0.10	0.93
i25	It would be difficult for me to stay away from home overnight	-0.13	0.10	1.14
i13	It is difficult to hide the fact that I wear a pouch	-0.22	0.10	0.92
i21	I worry that my condition is a burden to people close to me	-0.41	0.11	1.18
i22	I avoid close physical contact with my friends	-0.56	0.11	0.98
i16	My stoma makes it difficult for me to be with other people	-0.97	0.12	0.76
i8	I am afraid of meeting new people	-1.12	0.12	0.93
i18	I feel lonely even when I am with other people	-1.35	0.13	0.73
i23	I worry that my family feel awkward around me	-1.60	0.16	1.29

The index of person separation, PSEP, for the Stoma-QOL was 2.92, corresponding to a reliability of 0.90.

**Figure 2:**
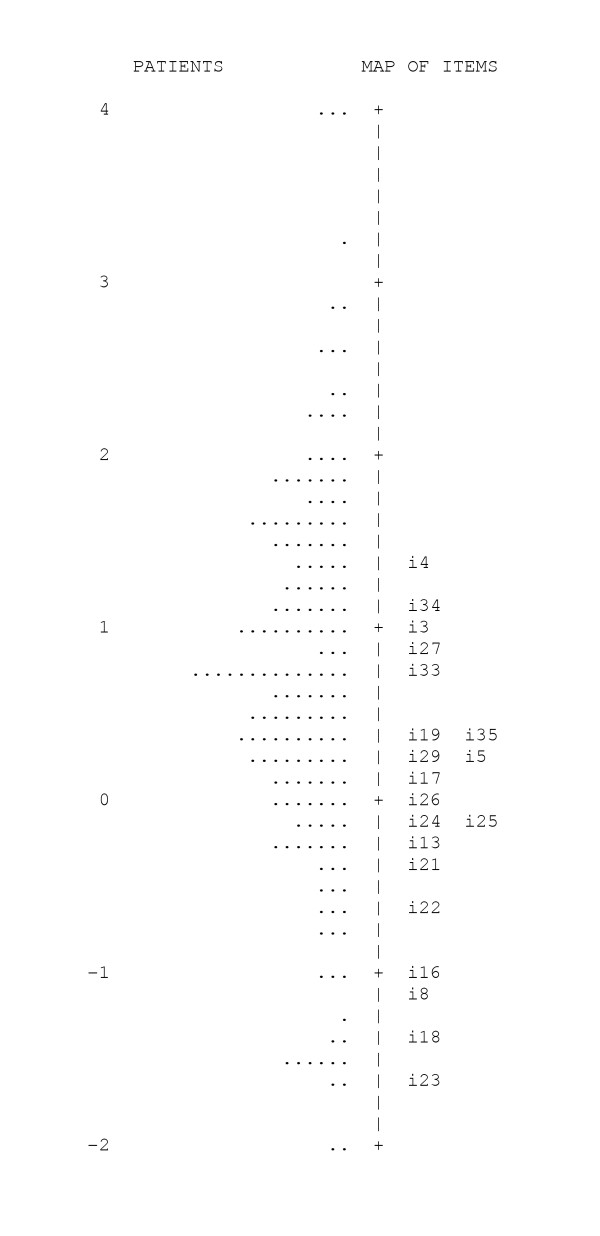
**Stoma-QOL Item Calibration of the items located within the spread of the person measures. **(logit scores, the measurement unit *common to both patients and items*, are displayed down the middle of the map; patient, represented by a single dot, are arranged within the scale from better (top) to worse (down) quality of life; items are identified by the item number)

In Figure 2, the calibrations, or "weights", of the items are located within the spread of the Stoma-QOL patient scores. The mean of the item calibrations was adopted by default as the 0 point. Item 26 was calculated as having that exact middle "weight", so it is located at the 0 point on the item-person map. Patients above a given item are likely not to indicate any concerns with it. Thus, the higher a patient is positioned on the map relative to the items of the test, the better in terms of quality of life. As an example, it can be seen that most of the patients indicated being "anxious when the pouch is full", so the majority of the patients are below the level of item 4; on the other hand, almost no-one had any concerns that their "family feel awkward around" them (item 23), so most patients were located above the calibration of item 23.

Rasch analysis was also performed on the categories used as response choices, indicating the 'distance' that separates the four response choices. These weights ranged from -0.94 to 1.66. The distance that separated response category "2. Sometimes" and "3. Rarely" (0.75) was found to be in the same range as the distance that separated "1. Always" and "2. Sometimes" (0.89) and "3. Rarely" and "4. Not at all" (0.96).

Item parameters by country also fitted to the Rasch model (Infit MNSQ<1.3) and had very similar item calibrations: ICC of the item calibrations by country was 0.81 (0.67–0.91 95% CI).

The secondary analysis according to classical test theory of the final reduced questionnaire gave the following results: The classical item discrimination index for the 20 items of the questionnaire ranged from 0.51 to 0.67, exceeding the minimum recommended value of 0.3. The mean response choice for each item (difficulty index) ranged from 2.11 to 3.60, which suggests that all items are moderately spread around the centre of the four response choices (1. Always; 2. Sometimes; 3. Rarely; 4. Not at all). Internal consistency reliability estimated as Cronbach's alpha for the Stoma-QOL was 0.92, exceeding the minimum recommended standard of 0.70.

In order to test whether a calculation of a global score from the Stoma-QOL is a valid measure, we inspected the scree plot in an Exploratory Factor Analysis. We found that a single component was an optimal solution with factor loadings in a single factor solution ranging from 0.53 to 0.72, accounting for 38% of the total variance.

Table 4 shows Spearman's correlation coefficients of the Stoma-QOL scores across times of administration of the questionnaire (T1 vs. T2 vs. T3) with all scores >0.88 (p < 0.01).

**Table 4:** Spearman's correlation coefficients of the Stoma-QOL scores across times of administration.

**Pair wise comparisons of times of administrations**	**Spearman**
1st vs. 2nd	r = 0.913
1st vs. 3rd	r = 0.881
2nd vs. 3rd	r = 0.946

Finally, to take into account each particular item calibration in the final Stoma-QOL scores, raw scores were transformed through Rasch modelling into a new score scale set to a minimum of 0 (Worst Quality of Life) and a maximum of 100 (Best Quality of Life) points. Overall mean on this scale was 58.5, with the lowest mean value in France, 53.8, significantly lower than Denmark with the highest mean value, 62.6 (p = 0.007).

Table 5 shows the scoring correspondence between the simple raw score of the four response choices to the 20 items of the Stoma-QOL and the final 0–100 score.

**Table 5:** Scoring Correspondence Between the Simple Raw Sum of the responses to the 20 items of the Stoma-QOL (responded in a scale: 1-Always; 2-Sometimes; 3-Rarely; 4-Not at all) and the Final 0–100 Score

**Raw Score (Simple Sum of 20 Items each scored from 1 to 4)**	**Final Score**	**Raw Score (Simple Sum of 20 Items each scored from 1 to 4)**	**Final Score**
**20**	0.00	**51**	53.47
**21**	11.54	**52**	54.13
**22**	18.48	**53**	54.88
**23**	22.70	**54**	55.53
**24**	25.80	**55**	56.19
**25**	28.24	**56**	56.85
**26**	30.30	**57**	57.50
**27**	32.08	**58**	58.16
**28**	33.58	**59**	58.91
**29**	34.99	**60**	59.57
**30**	36.30	**61**	60.32
**31**	37.52	**62**	60.98
**32**	38.65	**63**	61.73
**33**	39.68	**64**	62.48
**34**	40.62	**65**	63.32
**35**	41.56	**66**	64.17
**36**	42.50	**67**	65.01
**37**	43.34	**68**	65.85
**38**	44.18	**69**	66.79
**39**	45.03	**70**	67.82
**40**	45.78	**71**	68.95
**41**	46.53	**72**	70.08
**42**	47.28	**73**	71.39
**43**	48.03	**74**	72.89
**44**	48.78	**75**	74.58
**45**	49.44	**76**	76.55
**46**	50.19	**77**	79.17
**47**	50.84	**78**	82.83
**48**	51.50	**79**	89.02
**49**	52.16	**80**	100.00
**50**	52.81		

## Discussion

### Strengths and weaknesses of this study

Rooted in the lower three sections of Maslow's hierarchy of needs pyramid [10], Stoma-QOL items were generated by the asking the patients about their concerns over food, sleep, sexual activity, relations to family and close friends, and social relations to other than family and close friends. The subsequent Rasch analysis led to omission of the food domain, as items within this domain failed to contribute to construction of the global score. However, the final set of items within the remaining four domains of the Stoma-QOL successfully defined a meaningful measurement instrument with excellent psychometric properties with regard to validity and reliability.

It could be argued that a possible framing effect was present in the item generation phase. While we cannot rule out that some kind of biased selection of items may have been present during one or more steps of the development of the questionnaire, we find it important to underline that in our view selection of items is always a qualitative process, and thus somehow subjective. However, the resulting instrument has been subject to a quantitative analysis based on both classical test theory approaches and Rasch analysis, with fairly acceptable results.

Similarly, it could also be argued that the final four specific domains do not necessarily cover every relevant QOL issue specific to stoma patients, e.g. needs belonging in the top of Maslow's hierarchy of needs pyramid, such as "Ego needs" or "Self Actualisation" needs [10]. In fact, the relatively low level of variance explained by the Exploratory Factor Analysis (38%) indicates that there are other domains that impact on QOL, and this is an avenue for further research. However, the potential contribution of interventions, such as, e.g., stoma devices, to patients' QOL will by their nature primarily be correlated with improvements in the patients' basic functional status, and thus we consider these four domains to be adequate for the purpose. Furthermore, there are impracticalities associated with assessing an extensive number of domains in one instrument, e.g. increased length, complexity and time to completion.

In our opinion, the concept of responsiveness can be rejected as a separate measurement property of an evaluative instrument, a point of view supported by several authors [17-19], and for this reason responsiveness of the questionnaire was not tested. In opposition to a recent paper by Puhan et al. [20], we find that internal consistency coefficient adequately reflects an instrument's potential sensitivity to changes over time.

The statistically significant difference found between Denmark and France with regard to total Stoma-QOL scores, 8.8 points higher in Denmark than France, is notable, but it should be emphasised that the study was not designed to address this question – merely to show similar item calibrations between the countries.

For study-logistic reasons, only colostomy and ileostomy patients, but no urostomates, were included in the validation study. While we would predict that more similarities than differences exist between the different groups of stoma patients with regard to QOL issues, the use of Stoma-QOL in a population including urostomy patients would require testing for validity and reliability in the specific patient segment of urostomates.

Also, demographic data collection was limited to the main categories, colostomy and ileostomy, but for future studies it would be valuable to further subcategorise patients with regard to sigmoidostomies, transverse colostomies, loop stomas, etc., and perhaps also with regard to whether stoma surgery was performed on an elective or an acute basis. In the practical everyday surgical situation, the choice between performing the different types of stoma operation often has to be made more on the basis of experience than on the basis of evidence. If extended as suggested above, a tool such as Stoma-QOL would give the possibility of estimating potential differences between the different surgical techniques with regard to patients' expected post-operative quality of life. Used for this purpose, a tool such as Stoma-QOL could also contribute to providing more evidence with regard to variations in different age groups, in order to explore the importance of aiming to avoid stomas in those age groups particularly at risk of being affected by stoma-related reduced quality of life.

The patients included in this study were in general in a stable period or cured with a mean duration since stoma creation of more than seven years. A potential weakness of our study could therefore be a lack of sensitivity to QOL-related issues in patients whose stoma surgery took place recently. In the validation of the Ostomy Adjustment Scale, a small but significant relationship was found between global scores and the number of months elapsed since surgery [1]. Thus, for future studies of Stoma-QOL, we would recommend including a sufficient number of patients who have only recently undergone surgery to address these patients' specific concerns during their initial "adjustment process".

The full use of Stoma-QOL, for instance in a clinical trial, requires in principle that all 20 questions be answered. The reason is that each of the 20 questions will weigh with 1–4 points per question (1. Always; 2. Sometimes; 3. Rarely; 4. Not at all), and the summary patient score in the range of 20–80 will be converted to a global "0–100 score". In a clinical trial of an intervention, for instance a novel stoma pouch, some questions, more specifically i4, i34, i27, i35, i26 and i13 (Table 3), will be directly related to the intervention, while the remaining questions will be related, directly or indirectly, to the underlying condition (the stoma). In this situation it may be relevant to select only the questions related to the intervention and to omit the others. However, as the response burden of this 20-item questionnaire is already low (time to complete the questionnaire rarely exceeds 10 minutes), and in order to be able to calculate the overall QOL score, we generally recommend using the instrument in its full length.

### Comparison with other studies

The most important difference between Stoma-QOL and other instruments intended for stoma-patients is that the items in Stoma-QOL were generated by the primary source, the stoma patients themselves. The initial item generation was designed to get the patients to describe all their daily life concerns within the five pre-selected domains. This method, in our opinion, is preferable compared to item generation based on literature, experts and other second-hand sources. In contrast, the "Stoma Care Quality of Life Index" [6] was constructed as a modification of an existing tool, "QLI" for cancer patients, without involving the stoma patients in the item generation.

Another important difference between the Stoma-QOL and other more traditionally validated tools such as the "Stoma Care Quality of Life Index" [6] or the "Ostomy Adjustment Scale" [1] is that, as a result of the Rasch analysis, items are ordered from top to bottom according to the importance of the health problems (Table 3). The importance of each item is also reflected in the calculation of the total Stoma-QOL score, and thus the resulting measure, in our view, will be more meaningful to the clinician. For future studies, we plan to compare scores obtained with Stoma-QOL with scores obtained with one or more of the abovementioned instruments in order to investigate their degree of correlation, notwithstanding the differences in development and validation methods.

A specific instrument such as Stoma-QOL has its self-evident strengths as compared with generic instruments by virtue of its increased sensitivity to the unique problems related to a particular disease. As an example of obtaining a different outcome when using a specific as against a generic instrument in colorectal QOL research, a lower health-related QOL was found in a Danish study of Crohn's disease patients using a specific instrument [21], while, contrary to that result, another study using a generic instrument found health-related QOL to be at a level equalling that of the general population [22]. However, specific instruments also have limitations. In contrast to generic instruments, Stoma-QOL is not comprehensive outside its final four domains and cannot be used to compare with other conditions than stoma [23].

National versions of Stoma-QOL in English, Danish, German, French or Spanish and a User Manual in English can be downloaded upon registration at the website: 

## Conclusion

In conclusion, the metric properties of the Stoma-QOL questionnaire were assessed by means of Rasch analysis, taking into account the importance to the patients of the items, as well as the standard Classical Test Theory approaches, which assume that each item contributes equally to the total score. The importance weights of the 20 items of the Stoma-QOL spread out in a way that shows a coherent and meaningful direction, defining a variable of useful generality. Results also showed that the Stoma-QOL conforms to the Rasch model expectation of item weight invariance between national versions in different European countries. Given the adequacy of the metric properties of the Stoma-QOL suggested by the Rasch results as well as by the classical analysis, this study has shown the suitability of the instrument both for clinical practice and for clinical research.

## Authors' contributions

LP participated in the design of the study and performed the statistical analysis. HT planned and conducted the international collection of qualitative statements and supervised the translation including the field tests. KJ drafted the manuscript. All the authors read and approved the final manuscript.
